# Evaluation of Cytological Alterations of Oral Mucosa in Smokers and Waterpipe Users

**Published:** 2013-11-20

**Authors:** Safoura Seifi, Farideh Feizi, Mohammad Mehdizadeh, Soraya Khafri, Behrang Ahmadi

**Affiliations:** 1 Department of Oral and Maxillofacial Pathology, Cellular and Molecular Research Center, School of Dentistry, Babol University of Medical Sciences, Babol, Iran; 2 Department of Histology, Babol University of Medical Sciences, Babol, Iran; 3 Department of Oral and Maxillofacial Surgery, School of Dentistry, Babol University of Medical Sciences, Babol, Iran; 4 Department of Epidemiology, Babol University of Medical Sciences, Babol, Iran; 5 Babol University of Medical Sciences, Babol, Iran

**Keywords:** Cigarette Smoking, Waterpipe, Cytometric, Cytology, Oral Mucosa

## Abstract

**Objective:**

Oral mucosal epithelia of smokers and waterpipe users are more susceptible to malignant alterations. The aim of this study was morphometric evaluation of the effects of using waterpipe on normal oral mucosa.

**Materials and Methods:**

In a cross sectional study, cytologic smear samples from the following three different areas: buccal mucosa, lateral surface of the tongue, and floor of the mouth (right) were taken from 40 smokers, 40 waterpipe users, and 40 normal individuals. They were then stained using Papanicolaou staining technique. Quantitative cytologic alterations such as nuclear and cytoplasmic size, nuclear-cytoplasmic (N/C) ratio, Feret ratio (FR), percent of karriorhexis, vacuolization of cytoplasm, two or multilobed nuclei, inflammation, and candida were evaluated. Quantitative evaluation was performed using MoticPlus 2 software, and 50 cells in each slide were studied. Practitioners were matched with age and sex in three groups.

**Results:**

An increase in nuclear size, the N/C ratio, and F.R, while a decrease in cytoplasm size were observed in lateral surface of the tongue, buccal mucosa and floor of the mouth of smokers, waterpipe users and normal individuals, respectively (p≤0.001). No statistically significant differences were observed in percent of karriorhexis, vacuolization of cytoplasm, and two or multilobed nuclei in oral mucosa of smokers, waterpipe users (p=0.8), and normal individuals (p=0.9) in buccal mucosa, tongue, and mouth floor areas. However, the percentage of inflammation and candida in smokers (p<0.001) and waterpipe users (p=0.002) were higher than normal individuals.

**Conclusion:**

Smoking and using waterpipe are effective in creating some quantitative cytometric alterations in oral mucosa; however, smoking shows greater effect in the cytometric alterations than using waterpipe. Role of cytology in screening and detection of oral mucosa malignancies in smokers and waterpipe users needs further studies.

## Introduction

Squamous cell carcinoma of tongue is considered to be the most common oral malignant neoplasm (1). Cigarette, tobacco and waterpipe are among the most important etiologic factors of oral cancer and dangerous factors in dysplastic lesions (2,3).

Waterpipe is an instrument for smoking tobacco, which is popular in the Middle East and the Central Asia. To smoke a waterpipe, hot coals are kept in indirect contact with tobacco and the smoke is inhaled into the lungs (3). Many in the Middle East think that waterpipes are harmless with no addiction, while it is considered as a good substitute for cigarettes. Hence, using waterpipe is common in many cafes and entertainment centers. However, some studies have reported high levels of toxic substances, like carbon monoxide, heavy metals, and chemical carcinogenesis in waterpipe smoke (4,5). The first step in the treatment of cancer is the early diagnosis, especially in the high risk individuals (1). Genetic changes in epithelium happen in early stages of malignancy, while there are sometimes no clinical features in oral mucosa, which delays cancer diagnosis and causes irreparable damage (6). Cytology screening is the best method for early diagnosis of cancer because in long term studies of epithelium alterations, it is considered to be as a supplementary method which is fast, safe, non-invasive, inexpensive, with high sensitivity and without need of anesthesia, while it can be performed in form of either exfoliative cytology or brush cytology (7,8). However, the exfoliative cytology is not reliable method because of false positive and false negative responses (9).

Papanicolaou is the easiest and most common cytology technique for smear staining and is a routine method for diagnosis of malignant neoplasm of cervix (10). Cytometry is a technique for characterization and measurement of cells and cellular specifications like: nucleus size, cytoplasm size, nuclear-cytoplasmic ratio, aneuploidy and diploidy analysis of nucleus. The evaluations were performed using images from microscopic slides captured with attached camera system which are measured using special software (11). It seems that oral mucosa of smokers and waterpipe users are more susceptible to malignant changes varying in different oral areas (2). Most studies on smokers have only studied tissue specifications, but few of them have evaluated the cytological characteristics (10). Previous studies on quantitative cytomorphometry in oral mucosa of smokers, cocaine users, alcoholics, etc (12-14) have reported conflicting results. In the study by Ahmed et al. they have reported an increase in nuclear size, nuclearcytoplasmic (N/C) ratio and multi-lobed nuclei, while a decrease in size of cytoplasm in smokers as compared to non smokers (15). The study of Woyceichoski et al. (13) has also revealed an increase in cytoplasmic size and N/C ratio, while a decrease in size of cytoplasm in cocaine users as compared to the control group. In the study by Hosseini et al. they have reported more atypical changes in smokers in comparison to non smokers (16). To consider that no study has been conducted yet on waterpipe users, the aim of this study was to perform a quantitative cytomorphometric analysis in order to compare the smear samples of different normal mucosa from tongue, floor of the mouth, and buccal mucosa among smokers, waterpipe users, and normal individuals (non-smokers, nonwaterpipe users).

## Materials and Methods

The study was approved by the Ethics Committee of Babol University. In a cross sectional study, a total of 40 smokers, 40 waterpipe (hookah) users, and 40 normal individuals (nonsmokers, non-waterpipe users) were selected using easy non-probability sampling. Among smokers and waterpipe users, 38 individuals were from different cafes and entertainment centers of the city of Babol, Iran, while two individuals were dental students living in the dormitory of Babol University. The normal individuals were selected among students living in the boys’ dormitory of Babol University of Medical Sciences. All participants were male and were also age matched. To improve the accuracy of the study, age range was defined to be between 20 and 40 years old. The participants had no systemic disease and did not use alcohol. They did not have fixed or removable partial denture. The individuals who were exposed to cigarette smoke at home or work were excluded from the study (10). Among smokers, there were individuals smoking between 10 and 40 cigarettes per day for 6 to 15 years (17). The waterpipe smokers (hookah users) were individuals with habit of using waterpipe once to twice per week for 20-80 minutes during 3-5 years (3).

Normal individuals (nonsmokers, non-waterpipe users) were those who never had a history of smoking or using waterpipe. Three groups were match by age and sex (group matching). All participants signed a written informed consent form after the objective of the study was described to them by one of the researchers. The participants’ history of systemic conditions was also recorded. Clinical oral examinations were performed by an oral and maxillofacial pathologist. There was no oral lesion in oral mucosa of smokers, hookah users and healthy people. The participants also answered questions in a form regarding the number of cigarette consumption or the time and amount of waterpipe use. Before preparation of the cytologic smears, the participants were asked to rinse their mouth with saline solution. So, as to avoid staining of the mucoid material of saliva and food particles during staining process of slides, the sample areas were dried using a piece of sterile gauze. Then, from three anatomical areas, including floor of the mouth (right), postrolateral surface of the tongue (right), and anterior part of Stenson’s duct in buccal mucosa (right) were sampled separately using a disposable cytological brush (Cytobrush, PadtanTeb, Iran). The cytological brush was placed in contact with oral epithelium in the area. Using a constant medium pressure, the brush was spun 10-17 times, and the collected material was then smeared on a dried clean slide coded beforehand. Afterword, it was fixed immediately using Pothofix spray (95% ethanol; Padtan Tab, Tehran, Iran) sprayed at 25 cm distance from the surface with no more than two sprays. The written number on each slide for each participant could be followed using the number on the questionnaire form. The slides were stained within maximum of three days according to the Papanicolaou staining method. The following 10 steps were taken to stain the cytologic samples: i. placing in graded alcohol series (90˚, 70˚ and 50˚), ii. placing in distilled water, iii. staining with hematoxylin for 5-10 minutes, iv. placing in distilled water followed by acid alcohol (0.5%), v. exposing to distilled water and lithium carbonate, vi. washing with distilled water, vii. placing in graded alcohol series (50˚, 70˚, 90˚), viii. placing in orange solution for one minute, alcohol (95˚) and absolute alcohol, ix. fixed in xylene and x. finally mounting on glass and covering with cover glass. For quantitative cytomorphometric analysis, images were captured with attached camera system, transferred to Photoshop software, and analyzed using Motic- Plus 2 software (Micro-optic industral Group co. LTD). The images were captured at ×100 magnification using Olympus microscope (BX41, Tokyo, Japan). On average, 50 cells with strong staining were selected in each slide. To avoid mistakes in measurements, the cells were always count in one direction (left to right, top to bottom). Mean nuclear and cytoplasmic size in each cell, the N/C ratio, and Feret ratio (FR) (maximum to minimum nuclear diameter ratio) were then calculated. The results were expressed as mean ± SD (mm2).

### Quantitative cytomorphometric evaluation

In each cytologic slide, 50 cells in three microscopic fields were examined at ×100 magnification. The specifications of nucleus, such as cells number (percent) with two or multi-lobular nuclei, karyorrhexis, and vacuolization of cytoplasm in buccal mucosa, tongue, and mouth floor among smokers, waterpipe users and normal individuals were evaluated. The mean value of the results was expressed as percentage, while comparing among the three groups. The cytologic slides were evaluated for the existence of inflammation and candida in smokers, waterpipe users and normal individuals. The presence or absence of inflammation and candida was recorded, and the results were then reported as the percentage (number) of cytologic slides having inflammation or candida to the total number of slides in each group (40 cases).

### Statistical analyses

The results were then analyzed in SPSS (version 16, Chicago, Spss INC). The comparison among three groups was then performed using statistical analyses. Repeated measure, ANOVA and Tukey’s statistical tests were used to compare the mean value of nuclear size, cytoplasm size, the N/C ratio and FR among smokers, waterpipe users, and normal individuals in the following three areas: buccal mucosa, mouth floor, and tongue.

Percent of inflammation and candida among the three groups were compared using Mann-Whitney test.

## Results

A total of 120 individuals participated in this study including 40 smokers, 40 waterpipe users, and 40 normal individuals (control group). Mean age of participants was 30.32 ± 5.69, 30.15 ± 6.02, 30.3 ± 5.83 in smokers, waterpipe users, and the control group, respectively. There were no significant difference among three groups by age (p=0.1). All the participants were males.

### Cytomorphometric quantitative results

Tables 1, 2 and 3 demonstrate the highest values for the nuclear size, the nuclear-cytoplasmic ratio, and FR, while the lowest value of cytoplasm size in buccal mucosa (right), lateral surface of the tongue and floor of the mouth (right), respectively, in smokers, waterpipe users, and normal individuals (p<0.001 for all three).

**Table 1:** Mean values for nuclear size, cytoplasm size, the N/C ratio, and FR (big diameter of the nucleus/small diameter of the nucleus ratio) in smokers, waterpipe users, and normal individuals in baccal mucosa (right)


Groups	Nuclear size	Cytoplasm size	N/C ratio	FR

**Smokers**	398.598 ± 2236.2 ^c^	10010.4 ± 51969.7 ^c^	0.41 ± 0.003 ^c^	1.73± 0.02 ^c^
**Waterpipe users**	328.621 ± 2366.1 ^b^	10011.05 ± 73504.7 ^b^	0.32 ± 0.002 ^b^	1.33± 0.02 ^b^
**Control group**	247.560 ± 1731.5 ^a^	10101.1 ± 70686.7 ^a^	0.24 ± 0.001 ^a^	1.08± 0.009 ^a^


a, b and c; p<0/001, α=0.05.

**Table 2:** Mean values of nuclear size, cytoplasm size, the N/C ratio, and FR in smokers, waterpipe users, and normal individuals in lateral surface of the tongue (right)


Groups	Nuclear size	Cytoplasm size	N/C ratio	FR

**Smokers**	399.897 ± 205.6 ^c^	9467.231 ± 48293.7 ^c^	0.42 ± 0.001 ^c^	1.81 ± 0.02 ^c^
**Waterpipe users**	364.849 ± 201.7 ^b^	10271.2± 51640.2 ^b^	0.35 ± 0.004 ^b^	1.42 ± 0.02 ^b^
**Control group**	261.597 ± 155.8 ^a^	10926.5± 60746.11 ^a^	0.24 ± 0.002 ^a^	1.14 ± 0.02 ^a^


a, b and c; p<0/001, α=0.05.

**Table 3:** Mean values of nuclear size, cytoplasm size, the N/C ratio, and FR of smokers, waterpipe users, and normal individuals in floor of the mouth (right)


Groups	Nuclear size	Cytoplasm size	N/C ratio	FR

**Smokers**	384.251 ± 293.2 ^c^	9984.0 ± 7576.4 ^c^	0.38± 0.009 ^c^	1.6 ± 0.04 ^c^
**Waterpipe users**	314.476 ± 180.5 ^b^	10012.1 ± 59165.2 ^b^	0.31± 0.008 ^b^	1.2 ± 0.03 ^b^
**Control group**	247.324 ± 174.5 ^a^	10038.1 ± 68976.4 ^a^	0.24± 0.007 ^a^	1.01 ± 0.01 ^a^


a, b and c; p<0/001, α=0.05.

### The effect of smear location on quantitative variables

The difference in nuclear size, cytoplasm size, the nuclear-cytoplasmic ratio, and FR in tongue area, mouth floor and buccal mucosa of smokers was statistically significant (p<0.001 for all three).

The differences in the percentage of karyor-rhexis, number (percentage) of cells with two or multi- lobular nuclei, and vacuolization of cyto-plasm in three different areas (buccal mucosa, tongue, and mouth floor) among smokers, waterpipe users (p=0.8), and normal individuals were not statistically significant (p=0.9).

The difference in percent of inflammation in three different areas (buccal mucosa, tongue, and mouth floor) among smokers, waterpipe users, and normal individuals was statistically significant (p<0.001). In addition, the difference in percent of candida in mouth floor (p<0.001) and buccal mucosa (p=0.002) among smokers, waterpipe users and normal individuals was statistically significant (Table 4,Figes1,2).

**Fig 1:**
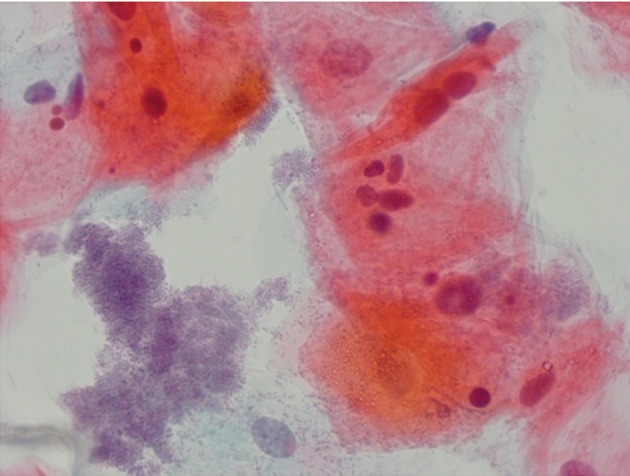
Cytologic sample of mouth floor stained by Papanicolaou method in a smoker showing multi-lobular nucleus and inflammation (×100).

**Fig 2:**
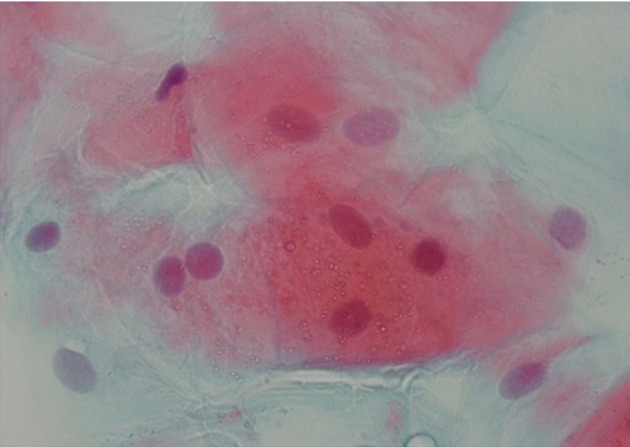
Cytologic sample of buccal mucosa stained by Papanicolaou method in a waterpipe user showing two-lobular nucleusand vacuolization of cytoplasm (×100).

**Table 4:** Comparison of nucleus state (number of cells with two or multi-lobular nuclei and karyorrhexis), vacuolization of cytoplasm, presence of inflammation or candida in cytologic smears of healthy oral mucosa among smokers, waterpipe users, and normal individuals


Groups	Smear location	Cells with two or multi-lobular nuclei	Karyorrhexis	Vacuolization of cytoplasm	Inflammation	Candida

**Smokers**	Baccal mucosa	42.9%	42.1%	30.8%	90%	75%
	Tongue	46.7%	40.9%	36%	100%	100%
	Mouth floor	46.7%	37.5%	32%	85%	87.5%
**waterpipe users**	Baccal mucosa	35.7%	36.2%	42.3%	70%	80%
	Tongue	33.3%	31.8%	44%	100%	100%
	Mouth floor	33.3%	33.3%	40%	100%	100%
**Normal individual**	Baccal mucosa	21.4%	31.6%	26.9%	45%	45%
	Tongue	20%	27.3%	20%	77.5%	77.5%
	Mouth floor	20%	29.2%	28%	77.5%	77.5%


## Discussion

Based on the results of this study, the biggest nuclear size, N/C ratio, FR and smallest cytoplasm size belong to smokers, waterpipe users and normal individuals, respectively. It is concluded that smoking and using waterpipe are effective in creating some quantitative cytometric alterations in oral mucosa, while our results confirmed that smoking has a greater effect than waterpipe user in this regard.

In microscopic study, one of the main symptoms of premalignant and malignant lesions is an increase in nuclear-cytoplasmic ratio (3,13), which we observed in the samples obtained from smoking and waterpipe, so it can be said that they are harmful.

Some studies have reported a similar risk of cancer in smokers and waterpipe users (17), while others have reported that waterpipe use is more harmful than smoking (18). It seems that the type of waterpipe, age, sex, size of sample studied, and even inclusion criteria for waterpipe users can affect the results of the study.

In a study by Hande and Chaudhary, they have performed a cytomorphometric analysis of buccal mucosa of tobacco chewers and reported an increase in the nuclear diameter and the ratio of nuclear diameter to cellular diameter, while a decrease in cytoplasm size in comparison with the control group (12).

However, in a study by Ogden et al. they have reported an increase in the nuclear diameter without a change in cytoplasm size in smokers as compared to the control group (19). Hilman and Kissin (20) have also reported an increase in the nuclear diameter and cytoplasm size in tobacco users. Hosseini et al. (16) have found more multilobed nuclei and pleomorphism in epithelial cells of smokers than non smokers. Regarding quantitative cytomorphometric alteration, the results of the current study is in agreement with the study of Hande and Chaudhary (12) and Hosseini Azimi et al. (16); however, our study showed different findings as compared to the results of Ogden et al. (19) and Hilman and Kissin (20).

In the current study, an increase in nuclear size in waterpipe users and smokers as compared to control group was observed. It seems that an increase in nuclear size is a kind of cell adaptation in response to the oral mucosa epithelium lesion. In other words, it is resulted from the increase of nuclear DNA content. Creating a cell irritation, smoking and waterpipe user facilitate aging process of oral mucosal cells. Epithelial cells of oral mucosa have a decreased turnover, so cells remain in cell cycle for longer periods resulting in a delayed cell division. As a result, proteins which are synthesized within the nucleus divide slowly, which in turn, it increases the nuclear size. The sizes of nuclear and cytoplasm decline following aging process as a result of degeneration of Golgi apparatus and endoplasmic reticulum in aged cells (21).

Inflammation is one of main factors affecting on nuclear and cytoplasm size, especially in smears prepared from young cells. Based on this information, we observed an increase in nuclear size, while a decline in cytoplasm size. However, it is not considered as cellular atypia (3,22). In our study, in order to decrease the effect of inflammation, cytologic smears were collected from the three different areas, including buccal mucosa, lateral surface of the tongue, and floor of the mouth. Moreover, cytological brush was used for both smear preparation and evaluation of the cells from the three different layers of epithelium (23). As a result, cells with different aging stages were present in sampling.

In the present study, opportunistic pathogens like candida was reported to be higher in smokers and waterpipe users compared to the control group.

In a study by Reis et al. (14) on buccal mucosa in alcohol users, they have showed an increase in carcinogenic cytologic changes, pyknosis, karyorrhexis in tongues of the alcohol users in comparison with the control group.

The reduction in cytoplasm size observed only in oral mucosa of smokers and waterpipe can be a result of dehydration which is a kind of cell adaptation in response to the decrease in fluids, especially saliva around the cell.

To consider that female hormones, such as estrogene and progesterone, influence growth and development of epithelial cells, and male hormones affect on bone metabolism and connective tissue matrix, cytomorphometric alterations or oral mucosa are certainly affected by hormones (24). The current study only included male individuals.

The question here is whether smear cytology location in oral mucosa can affect quantitative cytomorphometry. In this study, the location of smear preparation can affect the quantitative cytomorphometry result of epithelial cells of oral mucosa in smokers, waterpipe users and normal individuals. It appears that in comparison to buccal mucosa and floor of mouth, tongue has a higher exposure to carcinogen factors from cigarette and waterpipe smoke. The increase of N/C ratio in tongue area in some way confirms the result of the studies about tongue area as the most common site of squamous cell carcinoma (2).

In the study by Reis et al. on the effect of alcohol on cytologic smear in buccal mucosa and tongue, the increase in nuclear-cytoplasmic ratio only in tongue area was statistically significant between the two groups (14).

An increase in level of FR, expressing the nuclear shape, in smokers as compared to waterpipe users and healthy individuals was observed. The results of this study are in agreement with the results of the study by Goregen et al. (24). The higher values of FR for smokers in comparison to waterpipe users and normal individuals revealed that the nuclear shape was more oval.

It appears that the most important reason for the differences observed among the results of the studies is the lack of a specific method for the evaluation. Also, number of cytologic smears of practitioners, their age, location of cytological smear, timing between cytologic smear sampling and Papanicolaou staining technique, type of fixative, duration of fixation, and type of imaging software are effective in results. Further studies in oral smears are required in order to understand the cytology role in early detection of malignancies in oral mucosa of smoker and waterpipe users. The limitation of this study is our method sampling, which we suggested to be corrected for future studies.

## Conclusion

Smoking and waterpipe use are effective in creating some quantitative cytometric alterations in oral mucosa, while smoking shows greater effect than waterpipe use in this regard. Role of cytology in detection of oral mucosa malignancies in smokers and hookah users needs further studies.
